# Cetylpyridinium Bromide/Polyvinyl Chloride for Substantially Efficient Capture of Rare Earth Elements from Chloride Solution

**DOI:** 10.3390/polym14050954

**Published:** 2022-02-27

**Authors:** Eman M. Allam, Taysser A. Lashen, Saeyda A. Abou El-Enein, Mohamed A. Hassanin, Ahmed K. Sakr, Mohamed Y. Hanfi, M. I. Sayyed, Jamelah S. Al-Otaibi, Mohamed F. Cheira

**Affiliations:** 1 Nuclear Materials Authority, El Maadi, Cairo P.O. Box 530, Egypt; ah841873@ucf.edu (T.A.L.); mmsh236@yahoo.com (M.A.H.); akhchemist@gmail.com (A.K.S.); mokhamed.khanfi@urfu.ru (M.Y.H.); 2 Department of Chemistry, Faculty of Science, Menoufia University, Shebin El-Kom 32511, Egypt; dr.saeyda_elenein@yahoo.com; 3 Institute of Physics and Technology, Ural Federal University, St. Mira, 19, 620002 Yekaterinburg, Russia; 4 Department of Nuclear Medicine Research, Institute for Research and Medical Consultations (IRMC), Imam Abdulrahman Bin Faisal University (IAU), P.O. Box 1982, Dammam 31441, Saudi Arabia; mabualssayed@ut.edu.sa; 5 Department of Physics, Faculty of Science, Isra University, Amman 11622, Jordan; 6 Department of Chemistry, College of Science, Princess Nourah Bint Abdulrahman University, P.O. Box 84428, Riyadh 11671, Saudi Arabia; jsalotabi@pnu.edu.sa

**Keywords:** rare earth ions, cetylpyridinium bromide, polyvinylchloride, sorption, desorption

## Abstract

A new sorbent cetylpyridinium bromide/polyvinylchloride (CPB/PVC) was prepared and tested to extract rare earth elements (REEs) from their chloride solutions. It was identified by FTIR, TGA, SEM, EDX, and XRD. The impact of various factors such as pH, RE ion initial concentration, contacting time, and dose amount via sorption process was inspected. The optimum pH was 6.0, and the equilibrium contact time was reached at 60 min at 25 °C. The prepared adsorbent (CPB/PVC) uptake capacity was 182.6 mg/g. The adsorption of RE ions onto the CPB/PVC sorbent was found to fit the Langmuir isotherm as well as pseudo-second-order models well. In addition, the thermodynamic parameters of RE ion sorption were found to be exothermic and spontaneous. The desorption of RE ions from the loaded CPB/PVC sorbent was investigated. It was observed that the optimum desorption was achieved at 1.0 M HCl for 60 min contact time at ambient room temperature and a 1:60 solid: liquid phase ratio (S:L). As a result, the prepared CPB/PVC sorbent was recognized as a competitor sorbent for REEs.

## 1. Introduction

The rare earth elements (REEs) are the lanthanides series, scandium, and yttrium, except for promethium, all of which occur in nature. The REEs are found fixed in their minerals and act as the same chemical entity [1]. Rare earth elements are not found as individual compounds, but the mineral usually contains all the REEs with some enrichment of them by the cerium group or yttrium group. However, most REEs occur, in principle, only three ore minerals, namely, monazite, bastnasite (as a resource of the cerium group), and xenotime (as a source of the yttrium group) [2].

REEs have evolved into an essential aspect of current life in a wide range of products; thus, their recovery procedures now hold great consideration. The recovery of REEs is a complicated process that involves ore mining, mineral dressing, chemical upgrading, and refining. The crucial refining steps must result in the possible extraction of the REEs existing in the ore with the lowest possible cost [3,4,5,6].

Currently, there are great efforts to find innovative materials and technologies to extract RE ions from their solutions. Impregnation techniques have been developed as a substitute for ion exchange or solvent extraction as it overcomes the drawbacks of these techniques. These techniques depend on the alteration of solid support to extract the desired ions of their complex solutions. Two approaches were exploited for construction; the first method depends on the physical modification of solid support using a suitable reagent, and the second method comprises a chelating ligand toward the solid phase support. A variety of extraction techniques were evolved for selective extraction of RE ions using various solid-phase supports such as benzophenone, activated carbon, SiO_2_, titanium oxide, naphthalene, resins, clay, etc. [7,8,9,10].

REEs are recovered from ore materials, requiring some hydrometallurgical functions. Great efforts have been concentrated upon pristine materials to separate REEs. Impregnated resins were produced by altering solid supports for the separation of ions from complex matrices. Two methods were utilized to organize the solid set; one based on the physical modification of a proper solvent at the solid support. The second process implicates attaching the chelating complex to a support material. For individual REE extraction, various solid-extraction approaches have been conceived using solid supports of distinct types such as activated carbon, clays, SiO_2_, polymeric resins, titanium oxide, and naphthalene [10,11,12,13,14,15,16,17,18,19,20,21]. The extraction of lanthanides from positively acidic wastes has been proposed using a resin, which was developed by a dihexyl succinamic/chloromethylated polymer [22].

Nonionic Amberlite XAD-16 polymeric crosslinked with undiluted tributyl phosphate (TBP) was used to uptake Ce^4+^ ions from nitrate solution [23]. Mono aza dibenzo crown ether/Amberlite XAD-4 was employed for the removal of Sm^3+^, La^3+^, and Nd^3+^ [24]. A nonionic Amberlite XAD-4 polymeric anchored by vanillinsemicarbazone was used to separatee Ce^3+^ and La^3+^ [25]. Furthermore, La^3+^, along with Ce^3 +^ ions, were extracted with ethylhexyl phosphonic acid mono-ethylhexyl ester covered by polyvinyl alcohol and linked through divinyl sulfone or glutaraldehyde [26].

Rare earth ion extraction was also investigated using hexyl-ethyl-octyl-isopropylphosphonic acid/resin from HCl solutions [27]. Bis(2-ethylhexyl) phosphoric acid/polyethersulfone polymer was employed to separate rare earth ions from chloride, perchlorate, and sulfate media [28]. Moreover, bis(ethylhexyl)phosphoric acid/resin was utilized to separate Zn, Ca, Pr, Ce, Nd, Fe, Sm, and Al [29]. The rare earth ion separation was initiated by designing a polyethersulfone composite via phase inversion technique [30]. Extraction of La_2_O_3_ from monazite was performed in four steps: (a) extraction of lanthanum hydroxide using NaOH; (b) digestion with HNO_3_; (c) precipitation with NH_4_OH; and (d) calcination to La_2_O_3_ [31].

The recovery of REEs was examined using silica gel modified with diglycolamic acid [32]. A strong cation exchange resin (Amberlite 1R-120) was used for the adsorption of RE(III) ions from a standard and obtained leach liquor solution from phosphate ore by applying optimum leaching conditions; the optimum conditions for the loading of RE ions onto Amberlite 1R-120 were determined in a batch system [33]. Treated clay impregnated with m-aminophenol and amino-hydroxypyrazole was utilized to capture REEs [10].

In this study, polyvinyl chloride (PVC) was impregnated by cetylpyridinium bromide (CPB) to increase its sorption capacity. The physicochemical structure and properties of PVC and CPB/PVC sorbents were first characterized by SEM, XRD, EDX, FTIR, and TGA. Second, the REE sorption properties of these two materials were compared by studying the parameters that affected the sorption process such as pH, initial REE concentration, PVC and CPB/PVC dosage, and contact time. The uptake kinetics and sorption isotherms were also deliberated. Finally, the regeneration and recycling of the two sorbents were examined. 

## 2. Materials and Methods

### 2.1. Materials

The REE standard stock solution was prepared by dissolving a mixture of 0.2649 g of LaCl_3_, 0.3989 g of CeCl_3_·7H_2_O, 0.2632 g of PrCl_3_, 0.2606 g of NdCl_3_, 0.3639 g of SmCl_3_·6H_2_O, and 0.3294 g of YCl_3_ in 900 mL deionized water acidified by 15.0 mL concentrated HCl (36.5%) to prevent hydrolysis. These salts were supplied through B.D.H. Chem., Poole, England. The salts weights were equivalent to 150 mg/L for each individual La^3+^, Ce^3+^, Pr^3+^, Nd^3+^, Sm^3+^, or Y^3+^ ions. However, 900 mg of total RE ions were dissolved in 900 mL distilled water. Hence, RE ion concentration was equivalent to 1 mg/mL (1000 μg/mL = 1000 mg/L). Polyvinyl chloride ((CH_2_CHCl)_n_) was supplied by Sigma-Aldrich, St. Louis, MO, USA, it has a molecular weight (≈48,000 g/mol) and its molecular weight of the repeat unit was 62.5 g/mol. Additionally, cetylpyridinium bromide hydrate (CPB), arsenazo III, HCl, and NaOH were gained from Sigma-Aldrich.

A UV–Visible spectrophotometer (UV-2700i, Shimadzu, Kyoto, Japan) with 1.0 cm quartz cell covering the range of 200–1100 nm was used for the spectrophotometric analysis of total REEs by arsenazo III (indicator) at 650 nm as a control analysis for the adsorption process [34]. All pH measurements were carried out using a digital pH-meter, Inolab pH, Xylem Inc., Rye Brook, NY, USA, level 1.0, with an error of ±0.01 at ambient laboratory temperature. An inductively coupled plasma-optical emission spectrometer ICP-OES (Agilent 5800, Santa Clara, CA, USA) instrument was employed to measure the concentration of RE ions.

The sorbent’s crystal structure was examined by XRD (Empyrean, Malvern Panalytical, Almelo, The Netherlands). The sorbent’s morphology was scrutinized via scanning electron microscopy with energy dispersive X-ray analysis (SEM-EDX, Philips XL 30, Eindhoven, The Netherlands). The specific surface area, size of substances, and pore volume were measured using nitrogen sorption at 77 K (Nova 2000 series, Quantachrone Corporation, Boynton Beach, FL, USA). The functional groups of the studied sorbents were evaluated by Fourier transform infrared (FTIR) spectroscopy (IR Prestige-21, Shimadzu, Kyoto, Japan) using IR resolution software via the KBr method. The spectra were recorded in the range of 400–4000 cm^−1^, 4.0 cm^–1^ resolution, and 50 scans. A thermogravimetric analyzer (TGA 8000, Perkin Elmer, Waltham, MA, USA) was utilized to detect the thermal stability at 10 °C/min and temperature range of 25–900 °C.

### 2.2. Preparation of Cetylpyridinium Bromide Hydrate/Polyvinyl Chloride

**Scheme 1:**
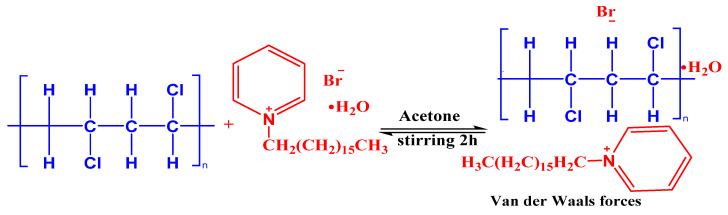
The suggested mechanism of cetylpyridinium bromide hydrate/PVC.

The dry method was utilized for the modification process. The impregnation conditions were optimized through a series of experiments. A total of 1.0 g cetylpyridinium bromide hydrate was dissolved in 25.0 mL acetone, and then it was added dropwise into a suspended solution of 10.0 g of polyvinyl chloride in 50.0 mL of acetone with stirring for 2.0 h at 25 °C until complete homogenization and dryness. The modified polyvinyl chloride with cetylpyridinium bromide hydrate was physically formed and dried at 60 °C (Scheme 1).

### 2.3. REE Sorption Studies

Many experiments were carried out to identify the applicable parameters affecting the REE extraction from the synthetic solution on both PVC and CPB/PVC adsorbents. The studied factors were solution pH, REE concentration, sorbent dosage, time of sorption, and temperature.

A series of experiments was studied by a different concentration of the REE synthetic solution with constant volume to fix the optimum parameters. The effect of pH on the sorption of REEs was examined with a pH value from 1.0 to 7.0. The pH in all experiments was adjusted by 0.2 M HCl as well as a 0.2 M NaOH solution. In contrast, the effects of sorbent quantity (10 to 90 mg) and contact time (5–120 min) were investigated. Moreover, different initial concentrations of rare earth ions were designated in 25–400 mg/L. The sorption uptake q_e_ (mg/g), efficiency (E, %), and distribution parameter (K_d_) were generated by the subsequent equations [35,36,37,38].

(1) $$q_{e}=\frac{\left(C_{o}-C_{e}\right)V}{m}$$

(2) $$E,\%=\left(\frac{C_{o}-C_{e}}{C_{o}}\right)\times 100$$

(3) $$K_{d}=\left(\frac{C_{o}-C_{e}}{C_{e}}\right)\times \frac{V}{m}$$

C_e_ and C_o_ (mg/L) are defined as the equilibrium and initial REE concentration, V (L) is solution volume, and m (g) represents the sorbent weight.

### 2.4. Desorption Studies

The rare earth ions loaded on PVC or CPB/PVC were exposed for stripping process to study the desorption aspects of the rare earth ions. The desorption methods were conducted on either 0.5 g of REE/PVC or REE/CPB/PVC using a 25.0 mL of various concentrations of HCl, NaCl, H_2_SO_4_, and HNO_3_ in the range from 0.1 to 3.0 M for a 60 min desorption time and then separated by filtration; the filtrate was analyzed to detect the concentration of eluting RE ions. The desorption parameters (the eluting type, the concentration of eluent, desorption time, and temperature) were investigated. After the REE-loaded adsorbent with treated with the eluting agent, it was rinsed carefully with deionized water to prepare it for recycling.

## 3. Results and Discussion

### 3.1. Depiction of Materials

#### 3.1.1. XRD Investigation

**Figure 1:**
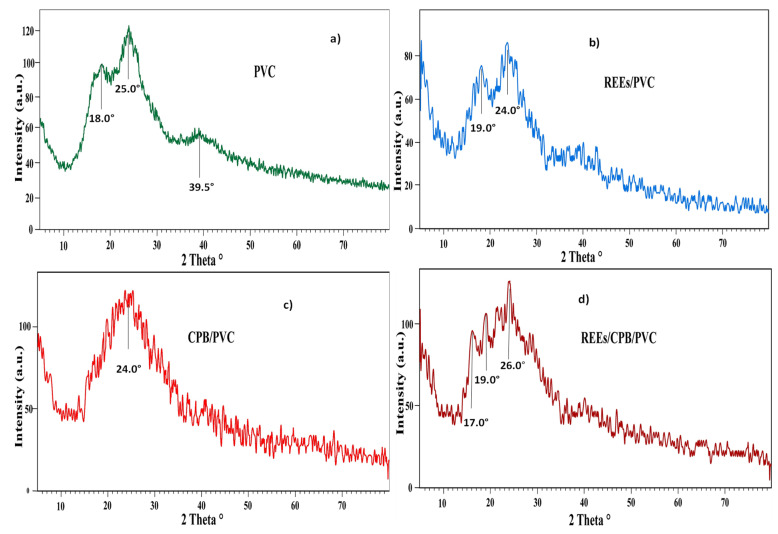
XRD analysis of (**a**) PVC, (**b**) REEs/PVC, (**c**) CPB/PVC, (**d**) REE/CPB/PVC.

The XRD spectra of PVC, CPB/PVC, REE/PVC, and REE/CPB/PVC are shown in Figure 1. The data in Figure 1a of the PVC show that the polymer exhibited two peaks at 2*θ* angles of 18.0° and 25.0° due to its amorphous nature [39]. However, the small peak at 2*θ* = 39.5° indicated the polymer semi-crystalline structure [40]. The other peaks were combined into one peak at 2*θ* = 24.0° for the CPB/PVC spectrum, and the peak at 2*θ* = 39.5° disappeared (Figure 1c). Consequently, it can be concluded that CPB was distributed into PVC to compose CPB/PVC.

The XRD of the REE/PVC spectrum, in Figure 1b was slightly broadened and shifted to 19.0° and 24.0°. Furthermore, the peak at 2*θ* = 39.5° in Figure 1a also disappeared due to the PVC polymer structure being amorphous. The data quantified that the rare earth ions were sorbed on the PVC surface. The XRD of REEs/CPB/PVC is shown in Figure 1d. From the XRD of CPB/PVC (Figure 1c), the peak position and peak shape of CPB/PVC at 2*θ* = 24.0° was enlarged and had a change in position to 2*θ* = 26.0°, and new peaks were gained at 17.0° and 19.0° for the XRD pattern of REE/CPB/PVC in Figure 1d, indicating that the REEs were adsorbed on CPB/PVC.

#### 3.1.2. SEM-EDX Investigations

**Figure 2:**
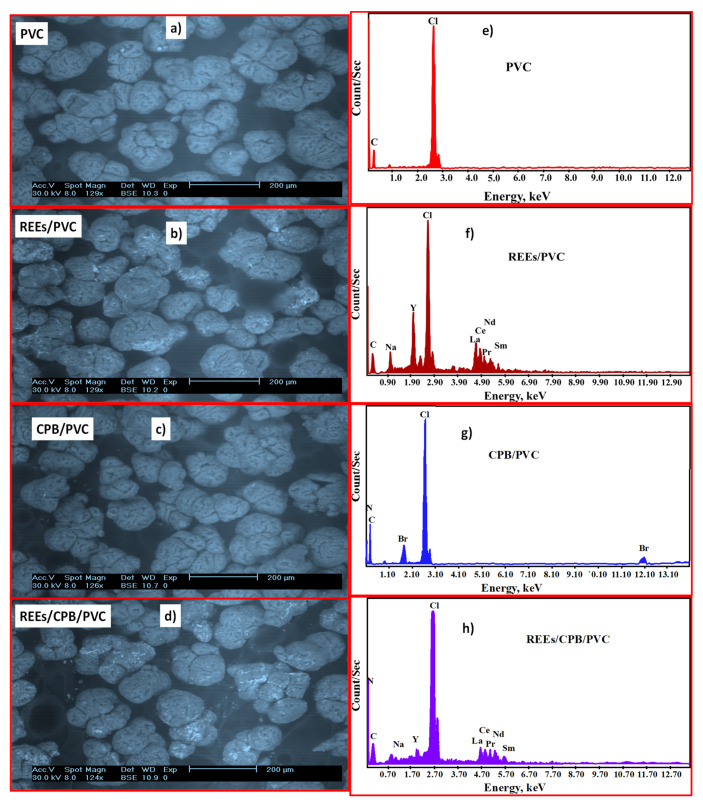
SEM pictures of (**a**) PVC, (**b**) REE/PVC, (**c**) CPB/PVC, (**d**) REE/CPB/PVC and EDX investigation of (**e**) PVC, (**f**) REE/PVC, (**g**) CPB/PVC, (**h**) REE/CPB/PVC.

Figure 2a illustrates that the PVC surface was approximately smooth, spotless, and had no fissures with minor drawbacks due to slight differences in the morphology of the tiny granules of PVC. At the same time, the CPB/PVC image was recognized with few aggregations with structures that seem to be a little globular and regular (Figure 2c). On the other hand, the loaded RE ions on PVC or CPB/PVC images in Figure 2b,d showed that the particles were agglomerated, and RE ions were observed as white spots on the surface of two sorbents. From the results, evident surface morphology changes in the REE/PVC or REE/CPB/PVC sorbents confirmed that the REE sorption onto the studied two sorbents was achieved.

The semi-quantitative EDX investigation of PVC along with CPB/PVC before and after REE sorption was premeditated. From the results, C and Cl bands were accessible in the PVC; furthermore, no additional bands were noticed (Figure 2e). In contrast, the CPB/PVC contained N, C, Cl, and Br peaks (Figure 2g). These results emphasize that the CPB/PVC was established and formed. Therefore, it can be concluded that CPB was distributed into PVC to form CPB/PVC due to the surface electrostatic interaction. After rare earth ion sorption, discrete peaks of some rare earth ions (Y, La, Ce, Pr, Nd, Sm) were found on the two sorbents (Figure 2f,h). The REE peaks were perceived and confirmed REE sorption on PVC and CPB/PVC.

#### 3.1.3. BET Surface Analysis

The Brunner–Emmett–Teller theory (BET) was utilized to examine the surface area of either solid or porous materials. It provides crucial information about their physical structure, as the area of the solid surface of the substance influences how that solid interacts with its surroundings. During either synthesis or processing, the surface area of a material can be changed. The surface area of a particle increases when pores are created within its interior by decomposition and dissolution as well as other physical or chemical means. A nitrogen adsorption–desorption analyzer was used to assess the specific surface area and the studied adsorbents’ pore size. The four samples were dried before analysis to 60 °C for 2.0 h.

**Figure 3:**
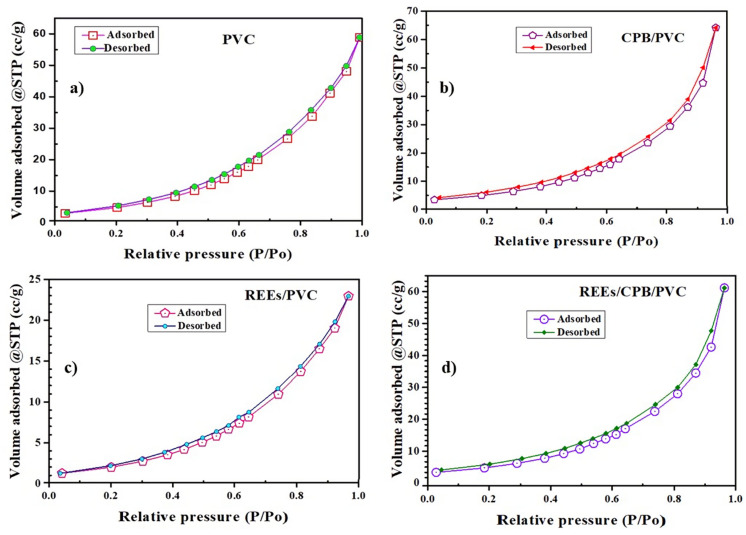
N_2_ sorption/desorption isotherm of (**a**) PVC, (**b**) CPB/PVC, (**c**) REE/PVC, and (**d**) REE/CPB/PVC.

Table 1 and Figure 3a illustrate that PVC surface area, pore size, and volume were 94.06 m^2^/g, 1.94 nm, and 0.091 cc/g. Moreover, the CPB/PVC surface area, pore size, and volume were 68.32 m^2^/g, 2.91 nm, and 0.099 cc/g (Figure 3b). With the addition of CPB to PVC, the surface area of CPB/PVC was decreased. From the result, it may be attributed that CPB was impregnated in PVC. After rare earth ion sorption, the surface area, pore size, and volume of the PVC or CPB/PVC sorbents were decreased because rare earth ions blocked the active sites (Figure 3c,d). The attained results exposed that rare earth ions were more strongly adsorbed on the CPB/PVC than the PVC due to CPB/PVC having more active spots than PVC.

#### 3.1.4. FTIR Investigation

**Figure 4:**
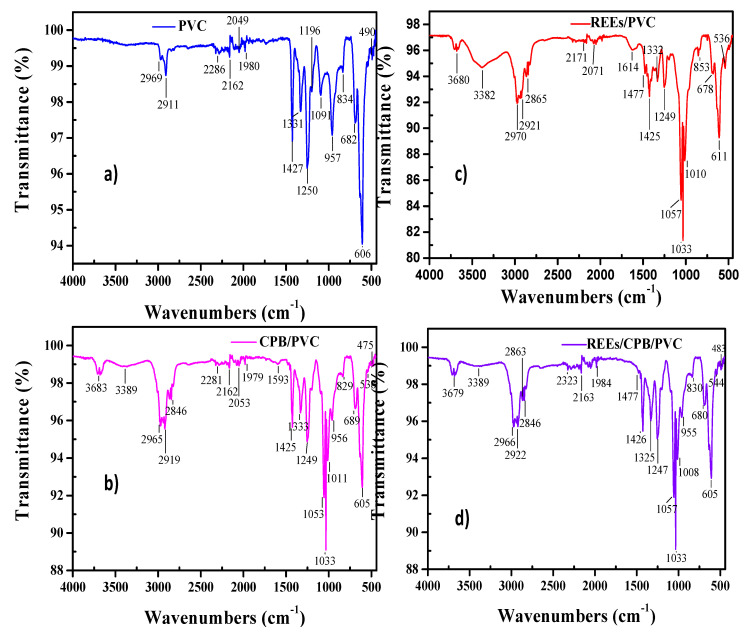
FTIR spectra of (**a**) PVC, (**b**) CPB/PVC, (**c**) REE/PVC, (**d**) REE/CPB/PVC.

The functional groups attached to the surface of the materials under study were recognized by FTIR spectroscopy [41]. The PVC spectrum in Figure 4a showed that two peaks at the 606 and 682 cm^–1^ were related to the C–Cl stretching mode, according to the polymer conformation structure and the locative position of the surrounding atoms according to C–Cl bonds [42]. The peaks at 2911–2969 cm^–1^ matched –CH_2_– and –CH groups. The assignments at 1250 and 1331 cm^–1^ indorsed –CH in the –CHCl group [43]. The intensity of the C–H band was also improved with the impregnation with cetylpyridinium bromide hydrate. Furthermore, the intensity of all of the C–Cl bands was increased.

The prepared cetylpyridinium bromide hydrate/polyvinyl chloride spectrum exhibited a band at 3389 cm^–1^ because of the O–H of hydration (Figure 4b). The vibrational band at 1593 cm^−1^ confirmed the C=C aromatic of the pyridinium group. The feature at 1425 cm^–1^ was predictable as O–H of the distortion peak of H_2_O. The comparatively broad features at 1250 and 1053 cm^–1^ indicate the C–N group. In addition, the two features at 605 and 689 cm^–1^ are due to C–Cl stretching vibration. Hence, the CPB was distributed into PVC to form CPB/PVC because of the electrostatic interaction at the surface (van der Waals forces) due to the presence of C–Cl, imino groups, and most of the peaks in CPB/PVC were shifted 5–10 cm^−1^. After REE sorption (Figure 4c,d), the vibration bands of PVC or CPB/PVC were reduced and shifted slightly to 3–11 cm^−1^, which could be due to the sorption of rare earth ions on the surface sorbents [44].

#### 3.1.5. Thermal Analysis

Thermogravimetric analysis (TGA) is a thermal analysis method for determining changes in both chemical and physical characteristics of the samples. TGA measurements are typically made as a function of temperature rise with a constant heating rate, or as a function of time with mass loss and a constant temperature. TGA measurements are typically made as a function of temperature rise with a constant heating rate, or as a function of time with mass loss with constant temperature. Physical phenomena such as second-order phase transitions, desorption, vaporization, and others can be considered using the TGA. In addition, chemical phenomena such as dehydration and decomposition can be detected. The TGA can easily determine the mass gain or loss of samples due to decomposition, loss of volatile compounds, oxidation, and degradation. Additionally, standardized TGA testing procedures can be used to determine the samples’ thermal stability in terms of their resistance to thermal decomposition or degradation [45].

**Figure 5:**
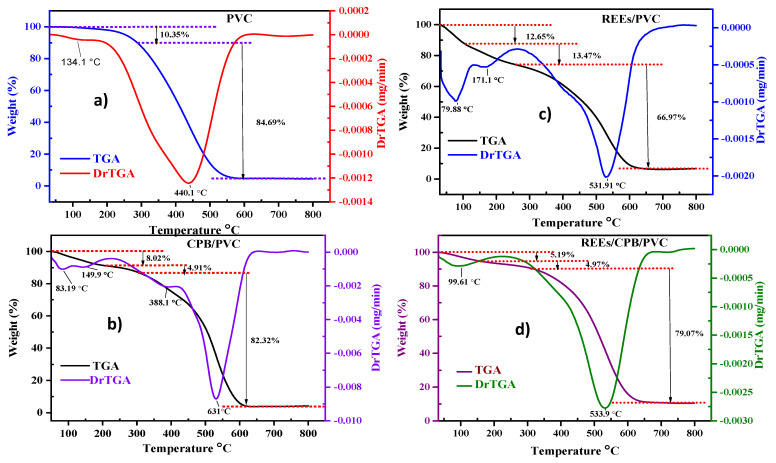
TGA analysis of (**a**) PVC, (**b**) CPB/PVC, (**c**) REEs/PVC, (**d**) REEs/CPB/PVC.

The TGA thermograms of PVC, CBP/PVC, REE/PVC, and REE/CBP/PVC are shown in Figure 5. The TGA curve of the PVC had two weight-loss steps with reference to the two thermal degradation steps that could be discerned in the TGA curve (Figure 5a). The first weight loss step occurred in the range of 220–332 °C and was related to removing the HCl consecutive reaction and forming a conjugated polyene structure. The second step appeared within the range of 445–535 °C due to the thermal degradation of the PVC carbon chain, which yields flammable volatiles [46,47].

The thermal stabilities of CPB/PVC and REE/CBP/PVC are also shown in Figure 5b,d, which had three weight loss steps. The first step that appeared at 115 °C was because of the loss of hydration H_2_O. The second step occurred at 255–345 °C due to eliminating HCl, HBr, and NH_3_ of thee CPB/PVC sorbent. The third step was within a 350–650 °C range due to the thermal degradation of the carbon chains, which generated ignitable volatiles. In contrast, the TGA of REE/PVC exposed three weight loss stages in Figure 5c. The first stage seemed at 110 °C was due to the loss of H_2_O related to REEs. The second stage occurred at 250 °C due to the removal HCl from the PVC sorbent. The third stage was within the 350–600 °C range because of the thermal decomposition of the carbon chains, which resulted in the production of flammable volatiles.

The remaining residues of PVC and CPB/PVC were 4.96 and 4.75%, respectively. Moreover, the residues of REE/PVC and REE/CBP/PVC were 6.91 and 10.77%, respectively. Hence, the residues of REE/PVC and REE/CBP/PVC were higher than the PVC and CBP/PVC due to the sorption and existence of REEs on the surface of the studied sorbents.

### 3.2. REE Sorption Studies

Batch techniques were executed to investigate the REE sorption on PVC or CPB/PVC as the sorbents from the RE chloride synthetic solution. These were conducted by contacting a mass of PVC or CPB/PVC with a fixed volume (50.0 mL) of RE ion solution. Several experiments on REE sorption efficiency were practically conducted to optimize the pH, sorbent amount, initial RE ion conc., time of contact, and temperature.

#### 3.2.1. Effect of Solution pH

**Figure 6:**
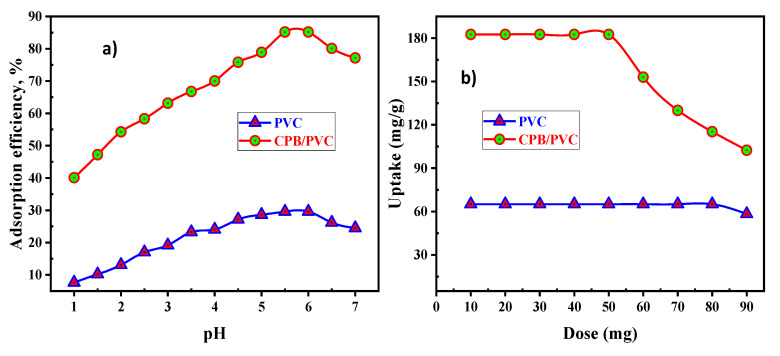
Effect of (**a**) pH and (**b**) sorbent dose on the REE sorption.

Numerous experiments were carried out with pH values from 1.0 to 7.0. In the meantime, the other procedure parameters were remained stable at a 50.0 mL solution of 200 mg/L REE, 50 mg PVC, or CPB/PVC dose for 30 min at ambient temperature. The gained data from Figure 6a showed that the REE sorption efficiencies were gradually increased from 7.64 and 40.1% to 29.6 and 85.2% for PVC and CPB/PVC, respectively, with an increase in the pH beyond 6.0. However, REE sorption efficiencies were decreased to 24.5% for PVC and 77.18% for CPB/PVC by increasing the pH from 6.0 to 7.0 values. Accordingly, a pH of 5.5–6.0 was determined as the optimal pH value to conduct the succeeding rare earth ion sorption experiments.

#### 3.2.2. Effect of Sorbent Dose

The effect of sorbent dosage on rare earth ion sorption uptake was studied. The obtained results in Figure 6b revealed that the uptakes of RE ions decreased from 65.12 and 182.6 mg/g to 58.33 and 102.44 mg/g with an increase in the dosage of sorbent material from 10 to 90 mg for PVC and CPB/PVC, respectively. Due to an increase in the surface area, more ion exchange sites exist for ion exchange procedures at a higher dose. Thus, the 50 mg dose was considered as a choice of sorbent dose in the subsequent experiments.

#### 3.2.3. Effect of Contact Time

**Figure 7:**
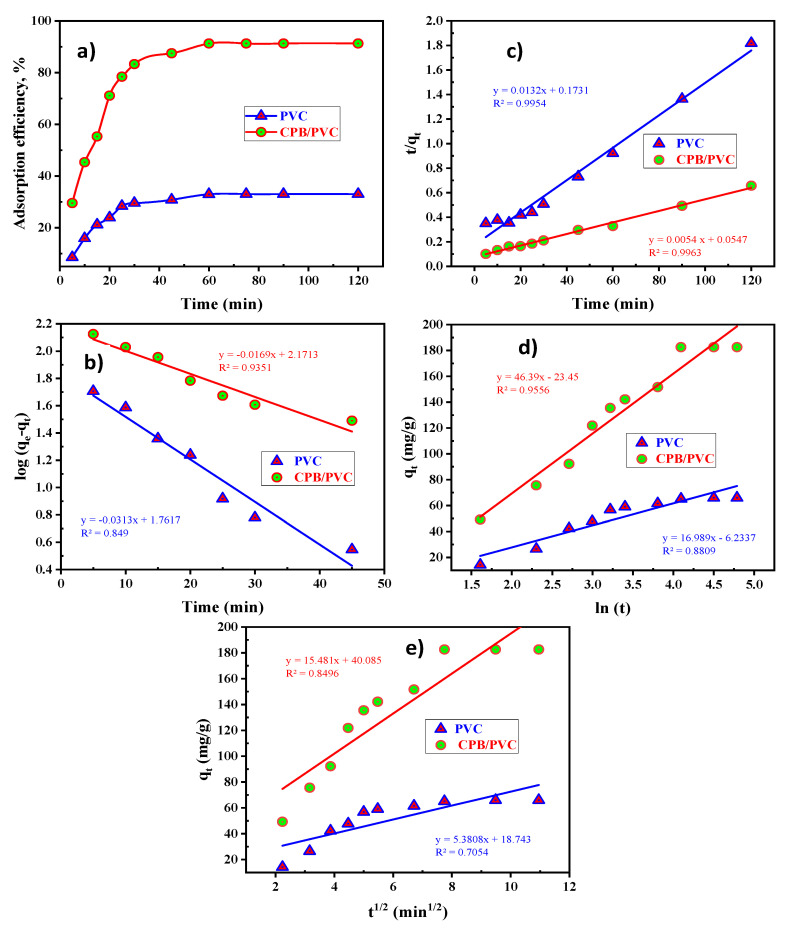
(**a**) Effect of contact time, (**b**) Pseudo-first-order, (**c**) Pseudo-second-order, (**d**) Elovich, (**e**) Intra-particle diffusion models of REE sorption on PVC and CPB/PVC.

The influence of agitation time on the RE ion sorption on either PVC or CPB/ PVC sorbents was studied from 5–120 min, although the other sorption factors were kept constant. As observed in Figure 7a, the sorption efficiency of REEs was increased gradually as the sorption time increased until it achieved equilibrium at 60 min, where its uptake was 65.12 mg/g for PVC and remained constant up to 120 min. The sorption uptake of RE ions gradually increased from 49.25 to 182.6 mg/g after 60 min using CPB/PVC, and then equilibrium was achieved.

##### Kinetic Characteristics

Different kinetic models were performed for experiential data to assume the RE ion sorption kinetics on PVC or CPB/PVC. The equation of the pseudo-first-order kinetic model was expressed as [48,49,50,51]:

(4) $$\log\left(q_{e}-q_{t}\right)=\log q_{e}-(\frac{k_{1}t}{2.303})$$

Both q_e_ and q_t_ (mg/g) are defined as the adsorbed amount of RE ions at equilibrium and at time t (min), and k_1_ (min^–1^) represents the pseudo-first-order rate constant. The results obtained from Figure 7b clarified that the R^2^ and q_e(cal)_ values for RE ion sorption on PVC or CPB/PVC did not follow a pseudo-first-order kinetic model.

The pseudo-second-order kinetic model was introduced by [52,53,54]:

(5) $$\frac{t}{q_{t}}=\frac{1}{k_{2}{\;q}_{e}^{2}}+\frac{t}{q_{e}}$$

where k_2_ refers to the pseudo-second-order rate constant (g/mg.min). From the data in Figure 7c, the plots exhibited straight lines for PVC and CPB/PVC with a R^2^ of about 0.995 and 0.996, respectively, nearly to unity. The sorption processes were right to the pseudo-second-order model, as observed in Table 2. Additionally, the proposed values (q_cal_) of adsorbed amounts at equilibrium were close to the practical sorption uptakes (q_exp_). The data show that the RE ion sorption upon PVC or CPB/PVC well obeyed the pseudo-second-order kinetic model.

The Elovich kinetic model [55,56,57] designates the chemical sorption characteristic, and is generally a linear equation according to the following:

(6) $$q_{t}=[\left(\frac{1}{\beta }\right)\ln\alpha \beta ]+\left[\left(\frac{1}{\beta }\right)\ln t\right]$$

where α corresponds to the initial sorption rate (mg/g min), while β denotes the Elovich constant (g/mg). From Figure 7d, R^2^ was 0.881 and 0.956 for PVC and CPB/PVC, respectively. Hence, it was seen that the Elovich model was not suitable for the sorption processes.

The intraparticle model describes the correlation between the RE ion uptake (q_t_) sorbed at a time (t) vs. the time square root (t^1/2^) linearly and can be expressed as [58]:

(7) $$q_{t}=k_{\mathrm{id}}t^{1/2}+I$$

where the k_id_ symbolizes the initial rate constant (mg/g.min^1/2^) of intra-particle diffusion; and I provides a perception of the boundary layer thickness.

The K_id_ values were estimated using the intra-particle diffusion plot’s slope, as seen in Figure 7e. The R^2^ values (0.705 and 0.849) showed that the intra-particle diffusion processes were not the rate-limiting stage of the PVC and CPB/PVC sorbents. The obtained parameters for this model are shown in Table 2. This means that intra-particle diffusion models would not be the rate-limiting stage.

#### 3.2.4. Effect of Initial REE Concentration

**Figure 8:**
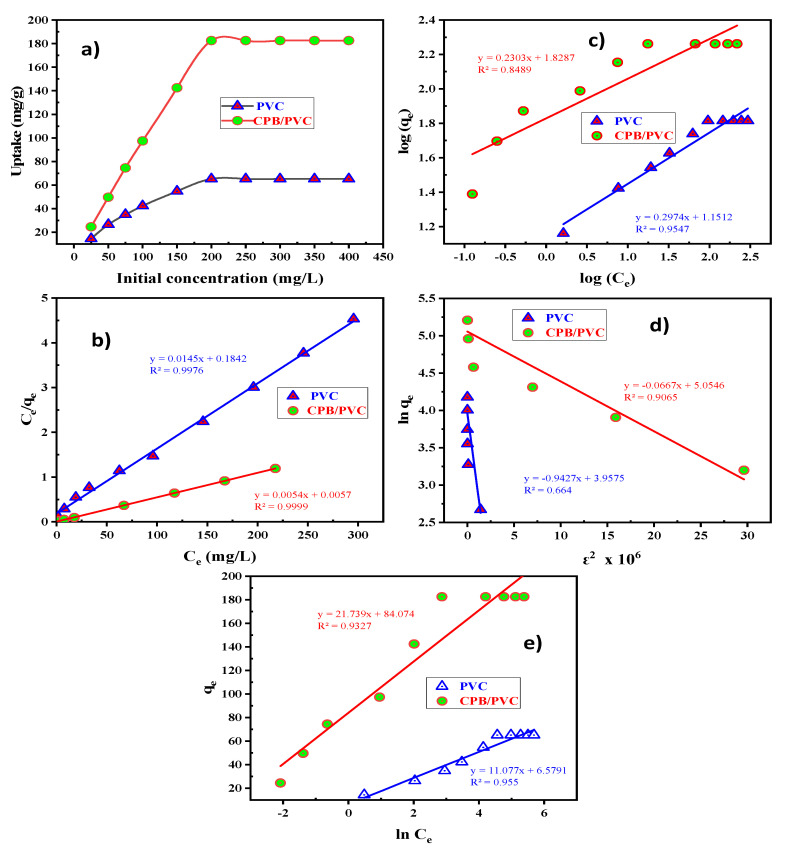
(**a**) Effect of initial REE concentration on REE uptake, (**b**) Langmuir, (**c**) Freundlich, (**d**) Dubinin and Radushkevich, and (**e**) Temkin isotherm models of REE sorption on PVC and CPB/PVC.

The attained results featured in Figure 8a show that the uptake (q_e_, mg/g) of RE ions increased with increasing initial RE ions concentration. The maximum uptake was achieved at 200 mg/L of RE ions. The maximal loading amount of RE ions on the PVC and CPB/PVC sorbents was 65.2 and 182.6 mg/g, respectively. The loaded REE uptake (q_e_) was still constant after 200 mg/L because the two sorbents reached their maximum loading capacities (saturation capacities). RE ions filled all active sites of the two sorbents.

##### Isotherm Characteristics

Studying the sorption isotherm models was necessary to discover the details of the sorption process such as the mechanism, surface properties, and other parameters that affect the sorption procedures.

In accordance with the Langmuir isotherm model, REs ion uptake occurs on a homogeneous surface through the monolayer of RE ions sorbed on the surface of PVC or CPB/PVC sorbents with steady sorption energy. In addition, there should be immobilization of the RE ions on the surface plane [59,60]. The linearized Langmuir isotherm model allowed for the intention of uptake capacity and Langmuir constant as follows:

(8) $$\frac{C_{e}}{q_{e}}=\frac{1}{q_{\max}\;b}+\frac{C_{e}}{q_{\max}}$$

where C_e_ (mg/L) refers to the equilibrium concentration in the aqueous layer, while q_e_ and q_max_ (mg/g) are the equilibrium and the maximum amount sorbed per unit weight of sorbent, respectively; and b is a constant correlated to the ability of the binding sites and the sorption energy (L/mg).

The plots of C_e_/q_e_ vs. C_e_ were tabulated in Table 3 and Figure 8b. From the obtained results, the calculated maximum capacities (68.96 and 185.18 mg/g) corresponded more to the practical data (65.13 and 182.60 mg/g), and the R^2^ was close to unity for both the PVC and CPB/PVC sorbents. Therefore, the working sorption process could obey the Langmuir sorption model.

The Freundlich isotherm model was applied to explore the RE ion sorption on the studied sorbent’s surface. It supposes that REE sorption takes place with various energetic layers of the active sites [61]. Equation (9) represents the Freundlich isotherm model:

(9) $$\log q_{e}=\log K_{f}+[\left(\frac{1}{n}\right)\log C_{e}]$$

where K_f_ (mg/g) represents the constant related to overall RE ion sorption uptake and n is related to surface heterogeneity. The constant n was deliberated at the slope of log q_e_ against the log C_e_ plot, as demonstrated in Figure 8c. K_f_ was calculated from the intercept simultaneously; the K_f_ (mg/g) values were less than the two sorbents’ experimental capacity of RE ions. The 1/n values were less than 1.0, demonstrating that RE ions preferred sorption by the PVC and CPB/PVC sorbents, while the R^2^ values were 0.955 and 0.849; this revealed that the Freundlich model also did not match the gained experimental results.

The Dubinin–Radushkevich (D–R) isotherm model offers a hypothetical distribution of energy sites. It is an excellent model to distinguish between physisorption and chemisorption [62,63]. It can be stated as follows:

(10) $$\ln q_{e}=\ln q_{D}-B_{D}\varepsilon^{2}$$

where q_D_ is the monolayer uptake (mg/g); B_D_ (mol^2^/kJ^2^) is related to the sorption energy constant; and ε represents the Polanyi potential correlated to the equilibrium concentration as the subsequent:

(11) $$\varepsilon =\mathrm{RT}\ln\left(1+\frac{1}{C_{e}}\right)$$

where T corresponds to the absolute temperature (K), and R is associated with the universal gas constant (8.314 J/mol.K). The following relation could calculate the free energy (E):

(12) $$Ε=\frac{1}{\sqrt{2{\;B}_{D}}}$$

where the q_D_ and B_D_ constants are inferred from the relation ln q_e_ vs. ε^2^ plots (Table 3 and Figure 4d). The sorption process is chemical if the magnitude of E is in the variety of 8.0–16.0 kJ/mol whereas the sorption process is physical if E is less than 8.0 kJ/mol [36,37]. The calculated E was less than 1.0 kJ/mol for RE ion sorption of the two studied sorbents, demonstrating that RE ions sorption proceeds via physisorption. However, (R^2^) values were 0.664 and 0.906 for PVC and CPB/PVC, respectively. Consequently, the (D–R) model did not suit the RE ion sorption on PVC and CPB/PVC.

The Temkin isotherm model postulated linear reduction in the sorption energy as the achievement rate of the sorption process toward the sorbent increases. Because of the sorbent–REEs interfaces, the sorption heat of all ions in the layer is regularly reduced with coverage. Moreover, the sorption process was described by a constant dispersal of binding energies, ready for maximum binding energy [64]. The Temkin isotherm could be indicated as follows:

(13) $$q_{e}=[\left(\frac{\mathrm{RT}}{b_{T}}\right)\ln K_{T}]+[\left(\frac{\mathrm{RT}}{b_{T}}\right)\ln C_{e}]$$

where b_T_ (kJ/mol) is defined as a constant interrelated to sorption heat; and K_T_ (L/g) points to the equilibrium binding constant. Both b_T_ and K_T_ were obtained by plotting q_e_ vs. ln C_e_ as shown in Table 3 and Figure 8e. From the obtained data, the values of R^2^ were 0.955 and 0.933 for the PVC and CPB/PVC sorbents, respectively. The data assumed that the sorption processes did not obey the Temkin isotherm model.

#### 3.2.5. Effect of Temperature

**Figure 9:**
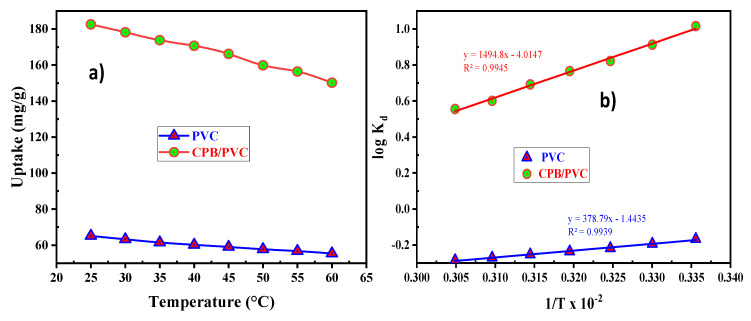
(**a**) Effect of temperature on REE uptake, and (**b**) log K_d_ vs. 1/T relation of REE sorption on PVC, and CBP/ PVC.

The influence of system temperature on RE ion sorption was examined from 25–60 °C. As demonstrated in Figure 9a, the REE sorption uptake of PVC and CPB/PVC diminished from 65.13 and 182.60 mg/g to 55.37 and 150.20 mg/g with an increase in the temperature due to the decomposition of the sorbent structure and decreased active sites. As a result, the ambient temperature was selected as the optimum temperature for RE ion sorption on the two sorbents.

##### Thermodynamic Studies

The effectiveness of temperature on the RE ions sorption process was examined to investigate the thermodynamic factors and identify the nature of the sorption process. It was detected that the sorption efficiencies of the two sorbents were reduced with an increase in temperature. The experimental data were utilized to determine the change in entropy (ΔS°), enthalpy (ΔH°), and thermodynamic parameters of RE ions sorption on PVC and CPB/PVC were described from successive equations [65,66,67].

(14) $$\log K_{d}=\frac{\Delta S^{{}^{\circ}}}{2.303R}-\frac{\Delta H^{{}^{\circ}}}{2.303\mathrm{RT}}$$

(15) $$\Delta G^{{}^{\circ}}=\Delta H^{{}^{\circ}}-T\Delta S^{{}^{\circ}}$$

where K_d_ refers to the RE ion sorption constant (L/g); ΔH° is the enthalpy changes (kJ/mol); ΔS° symbolizes for the changes in the entropy of RE ions sorption (J/mol.K); ΔG° corresponds to Gibbs free energy (kJ/mol); and T represents the absolute temperature (K). Both ΔS° and ΔH° can be gained from the intercept and slope of logK_d_ vs. 1/T plot (Figure 9b). The ΔH°, ΔS°, and ΔG° values for the two sorbents can be found in Table 4. The positive ΔG° values of the PVC sorbent proved that the RE ion sorption process is non-spontaneous. Nonetheless, the negative ΔG° value verified the spontaneous behavior of RE ion sorption on the CBP/PVC. Furthermore, the process was favorable for forming an electrostatic interaction between RE ions and CBP/PVC sorbent.

The negative ΔH° values might propose the exothermic sorption process. Additionally, the negative ΔS° values indicated the probability of the sorption processes and the decrease in randomness at the connection point between the sorbent/sorbate during the sorption processes of RE ions on PVC and CPB/PVC.

### 3.3. Desorption Studies

The desorption of metal ions is decisive in designing a sorption system; this step is intended to improve the desired metal ion concentration for final recovery. Moreover, this process is also vital in testing the recyclability of the sorbent, which is a significant feature for economic competitiveness.

#### 3.3.1. Type of Eluting Agent

**Figure 10:**
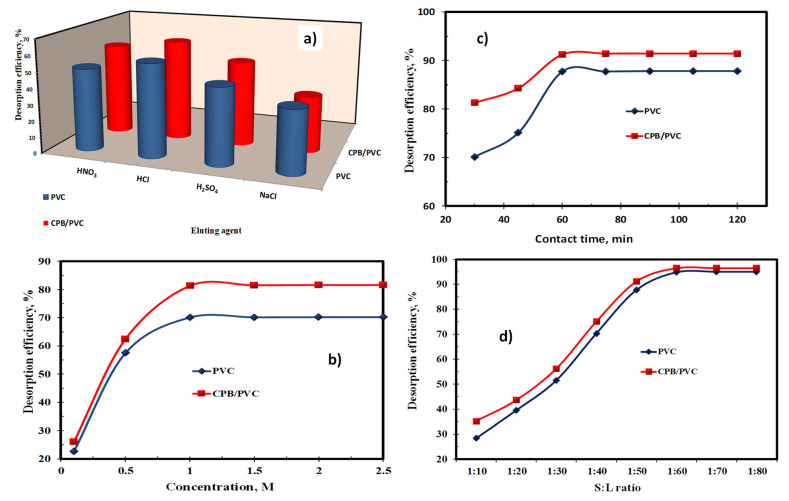
Effect of (**a**) eluting agents, (**b**) HCl concentration, (**c**) desorption time, and (**d**) S:L ratio upon REE desorption efficiency for the PVC and CBP/PVC sorbents.

Impact of eluting agents on the desorption process of rare earth ions from the loaded PVC and CPB/PVC was explored through the batch technique by shaking different eluting agents (NaCl, HCl, HNO_3_, and H_2_SO_4_) while the other parameters were still constant at 0.5 M of the eluting agent at a 1:50 S:L phase ratio (0.1 g of sorbent and 5.0 mL of eluting agent) for 30 min at ambient temperature. As seen in Figure 10a, it was observed that the desorption efficiency of RE ions from loaded PVC or CBP/PVC using 0.5 M hydrochloric acid reached maximum desorption efficiencies, attaining 57.5 and 62.4%, respectively. Therefore, it was concluded that hydrochloric acid can be used to desorb REEs quantitatively from the surfaces of the two sorbents.

#### 3.3.2. HCl Concentration

The REE stripping from their loaded sorbents was studied using a concentration range of HCl from 0.1–2.5 M. The other factors were kept constant using 0.1 g loaded sorbents and 5.0 mL of the eluting solution for 30 min at 25 °C room temperature. The gained data represented in Figure 10b illustrates that the desorption efficacy of RE ions improved with an increase in the concentration of HCl up to 1.0 M. In contrast, the optimum values of desorption were achieved at 70.1 and 81.3% for the PVC and CBP/PVC sorbents, respectively. Consequently, it can be concluded that 1.0 M HCl is recommended for the elution process of rare earth ions.

#### 3.3.3. Desorption Time

The influence of desorption time on the RE ion desorption was examined. For this purpose, 0.1 g REE/sorbent (REE/PVC, or REE/CPB/PVC) were mixed with 5.0 mL of 1.0 M HCl by different contact times ranging from 30 to 120 min at ambient temperature. According to the data represented in Figure 10c, it was clear that 60 min of contact time was required for the maximum RE ion desorption efficiencies at 87.7% and 91.2% for the PVC and CPB/PVC sorbents, respectively.

#### 3.3.4. S:L Phase Ratio

The influence of S:L phase ratio on REE desorption from their loaded sorbents was examined in the range of 1:10 to 1:80 to investigate the minimum volume of eluting agent required. It was observed that the rare earth ion desorption efficiency was improved by raising the S:L ratio to 1:60; following this, the REE desorption efficiencies were nearly constant at 94.8 and 96.5% for the PVC and CBP/PVC sorbents, respectively (Figure 10d). Therefore, the 1:60 S:L ratio of the studied sorbents was optimized for the subsequent experiments.

### 3.4. Regeneration

The PVC or CBP/PVC were regenerated by 1.0 M of HCl and 1:60 S:L ratio at room temperature for 60 min contact time to be recycled and reused to another sorption experiment. The sorption and desorption processes were recurrent until the desorption efficiency was decreased from 94.8 and 96.5% to 76.0 and 78.0% for the PVC and CBP/PVC, respectively, after eight consecutive sequences. It was decided that there was good sorption constancy of the two considered sorbents for REE recovery.

### 3.5. Case Study

The studied sample consisted of lamprophyre dikes collected from the area of Abu Rusheid in the South-Eastern Desert of Egypt. Lamprophyre dikes are mainly comprised of kaolinite, muscovite, K-feldspars, quartz, biotite, and plagioclases [68]. The sample of lamprophyre dikes was wholly analyzed by suitable techniques to specify major as well as trace ions. The data clearly show that analysis of SiO_2_, Fe_2_O_3_, Al_2_O_3_, K_2_O, CaO, and P_2_O_5_ assayed 46.3, 15.36, 16.8, 2.8, 2.3, and 1.02%, respectively. The sample had 3274 mg/kg of yttrium and 400 mg/kg of uranium. Furthermore, individual rare earth ions were analyzed using the ICP-OES technique (Table 5) [69].

The leach liquor of REEs was prepared by treating 2.0 kg of a milled sample of lamprophyre dike with 8.0 L of 3.0 M HCl for 3.0 h at ambient temperature [69]. The residue was then filtered, and the leach solution was analyzed, as shown in Table 6 and Table 7.

After preparing and analyzing leach liquor according to the studied optimum condition, PVC and CBP/PVC were used to carefully remove rare earth ions from the treated leachate under optimal conditions. Furthermore, the sorption efficiencies of 48.1 and 87.5% were obtained for PVC and CPB/PVC, respectively. The REE sorption efficiency from leach liquor was slightly lower than the maximum sorption efficiency of PVC and CBP/PVC sorbents from the pure synthetic solution due to impurities in leach liquor.

**Figure 11:**
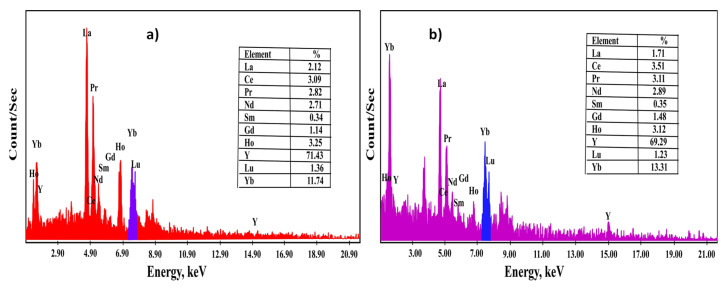
(**a**) The environmental scanning electron microscope of rare earth oxalate from REEs/PVC. (**b**) The environmental scanning electron microscope of rare earth oxalate from REEs/CBP/PVC.

Loaded sorbents (REE/PVC and REE/CPB/PVC) were subjected to desorption under the studied optimum conditions (1.0 M HCl, 1:60 S:L ratio, 60 min desorption time, 25 °C). Desorption efficiencies of 94.8 and 96.5% of REE/PVC and REE/CPB/PVC were obtained, respectively. After desorption and preconcentration, oxalic acid was used to precipitate RE ions as RE oxalates. The RE ions were precipitated using 20.0% oxalic acid (H_2_C_2_O_4_) as a rare earth oxalate precipitate [70,71]. The obtained precipitates of the two sorbents were analyzed and confirmed using SEM-EDX along with ICP-OES to detect their individual REEs distribution, as presented in Table 8 and Figure 11.

## 4. Conclusions

Polyvinyl chloride was treated with cetylpyridinium bromide to obtain a highly efficient, low-cost sorbent with outstanding results for RE ion sorption. The PVC and CPB/PVC were utilized to enhance the RE ion uptake from a chloride solution. The optimum sorption conditions were pH 6.0, 60 min equilibrium time, and 25 °C. The uptake of RE ions upon PVC and CPB/PVC were 65.13 and 182.6 mg/g, respectively. The RE ion sorption well obeyed the Langmuir isotherm model, and the pseudo-second-order model proved that the sorption occurred through chemical interaction. Additionally, the thermodynamic studies proved that the sorption of RE ions was exothermic and spontaneous for the CPB/PVC sorbent, and the maximum elution of RE ions from the loaded sorbent was obtained at the 1:60 S:L ratio and 1.0 M HCl for 60 min at ambient temperature. Thus, the CPB/PVC sorbent was recognized as a competitive sorbent for REE recovery.

## Figures and Tables

**Table 1:** Surface area, porosity, and pore volume of PVC and CPB/PVC previously and after rare earth ion sorption.

Materials	S_BET_, m^2^/g	Pore Size, nm	Pore Volume, cc/g
PVC	94.06	1.94	0.091
CPB/PVC	68.32	2.91	0.099
REE/PVC	39.09	1.82	0.035
REE/CPB/PVC	65.19	2.88	0.095

**Table 2:** Kinetic factors for REE sorption upon PVC and CPB/PVC sorbents.

Kinetic Models	Parameters	PVC	CPB/PVC
Pseudo-first-order	q_e_ (mg/g)	57.77	148.35
	k_1_ (1/min)	0.075	0.039
	R^2^	0.85	0.93
Pseudo-second-order	q_e_ (mg/g)	75.75	185.18
	K_2_ (g/md.min)	1.01 × 10^–3^	5.33 × 10^–4^
	R^2^	0.95	0.996
Elovich	α (mg/g.min)	11.73	27.13
	β (g/mg)	0.0589	0.022
	R^2^	0.881	0.956
Intra-particle diffusion	k_id_ (mg/g.min^1/2^)	5.38	15.48
	I (mg/g)	18.74	40.08
	R^2^	0.705	0.849
Practical capacity	q_exp_ (mg/g)	65.13	182.60

**Table 3:** Isotherm restrictions of isotherm modeling for REE sorption upon the PVC and CPB/PVC sorbents.

Isotherm Models	Parameters	PVC	CPB/PVC
Langmuir	q_max_ (mg/g)	68.96	185.18
	b (L/mg)	0.079	0.947
	R^2^	0.997	0.999
Freundlich	K_f_ (mg/g)	14.16	67.41
	1/n (mg.min/g)	0.297	0.230
	R^2^	0.955	0.849
D−R	q_D_ (mg/g.)	52.33	156.74
	B_D_ (mol^2^/kJ^2^)	0.943	0.067
	E (kJ/mol)	0.728	2.738
	R^2^	0.664	0.906
Temkin	b_T_ (J/mol)	223.67	113.97
	K_T_ (L/g)	1.81	47.79
	R^2^	0.955	0933
Practical capacity	q_exp_ (mg/g)	65.13	182.60

**Table 4:** Thermodynamic parameters of REE sorption on the PVC and CPB/PVC.

Parameters	Temperature, K	PVC	CPB/PVC
ΔG°, kJ/mol	298.0	0.9748	–5.7038
	303.0	1.1128	–5.3193
	308.0	1.2508	–4.9348
	313.0	1.3888	–4.5503
	318.0	1.5268	–4.1658
	323.0	1.6648	–3.7813
	328.0	1.8028	–3.3968
	333.0	1.9408	–3.0123
ΔH°, kJ/mol		–7.25	–28.62
ΔS°, kJ/mol.K		–0.0276	–0.0769

**Table 5:** Chemical analysis of lamprophyre dike ore.

Major Oxides	Wt., %	Metal Ions	mg/kg	REE Ions	mg/kg
SiO_2_	46.30	U^6+^	400	Y^3+^	3274
Al_2_O_3_	16.80	Ba^2+^	167	Ce^3+^	160
Fe_2_O_3_	15.36	Pb^2+^	596	La^3+^	90
TiO_2_	3.40	V^5+^	240	Nd^3+^	143
K_2_O	2.80	Cu^2+^	367	Sm^3+^	26
Na_2_O	0.48	Ni^2+^	86.7	Gd^3+^	60
CaO	2.30	Cd^2+^	49.5	Ho^3+^	171
MnO	0.65	Zn^2+^	6348	Er^3+^	145
P_2_O_5_	1.02	Th^4+^	39	Yb^3+^	550
MgO	0.06			Lu^3+^	65
LOI*	8.75			Pr^3+^	170
Total	97.92				

LOI*: Loss of ignition (1000 °C).

**Table 6:** Chemical investigation of metal ions in the leach liquor.

Metal Ions	Conc. (g/L)	Metal Ions	Conc. (mg/L)
Si^4+^	1.35	U^6+^	94.0
A1^3+^	2.34	REEs	1140.0
Ti^4+^	0.32	Ba^2+^	44.0
Fe^3+^	3.57	Pb^2+^	103.0
Mn^2+^	0.50	V^5+^	51.0
Mg^2+^	0.31	Cu^2+^	85.0
Ca^2+^	0.67	Ni^2+^	19.0
K^+^	0.73	Th^4+^	5.0
Na^+^	0.99	Zn^2+^	620.0
P^5+^	0.12		

**Table 7:** Chemical investigation of RE ions in the leach liquor.

Metal Ions	Conc. (mg/L)	Metal Ions	Conc. (mg/L)
La^3+^	21.0	Tb^3+^	0.0
Ce^3+^	37.0	Dy^3+^	0.0
Pr^3+^	40.0	Ho^3+^	40.0
Nd^3+^	34.0	Er^3+^	34.0
Sm^3+^	6.0	Tm^3+^	0.0
Eu^3+^	0.0	Yb^3+^	129.0
Gd^3+^	14.0	Lu^3+^	16.0
Y^3+^	769.0		

**Table 8:** Chemical analysis of RE oxalate products obtained from REEs loaded on two sorbents using ICP-OES.

Metal Ions	Conc. (mg/kg)	
	PVC	CPB/PVC
La^3+^	2415.394	7171.722
Ce^3+^	4546.624	13,499.71
Pr^3+^	4830.788	14,343.44
Nd^3+^	4120.378	12,234.11
Sm^3+^	568.328	1687.464
Gd^3+^	1776.025	5273.325
Ho^3+^	4901.829	14,554.38
Yb^3+^	17,476.09	51,889.52
Lu^3+^	1989.148	5906.124
Y^3+^	99,457.4	295,306.2
Total	142,082	421,866

## Data Availability

Not applicable.
